# A rare Bochdalek hernia in an adult: a case report

**DOI:** 10.1186/s12893-021-01357-2

**Published:** 2021-10-12

**Authors:** Yi-Min Gu, Xiao-Yang Li, Wen-Ping Wang, Long-Qi Chen

**Affiliations:** Department of Thoracic Surgery, West China Hospital, Sichuan University, No. 37, Guoxue Alley, Chengdu, 610041 China

**Keywords:** Bochdalek hernia, Congenital diaphragmatic hernia, Adult, Chest pain, Anomaly of intestinal rotation, Patch repair

## Abstract

**Background:**

Symptomatic Bochdalek hernias are found mainly in infants in respiratory distress and occur rarely in adults.

**Case presentation:**

We report a rare case of Bochdalek hernia associated with developmental abnormalities in an adult who exhibited acute chest pain and dyspnea on exertion.

**Conclusions:**

This case highlights the importance of the differential diagnosis of acute left-sided chest pain and antenatal examination.

## Background

Bochdalek hernia (BH) is one of the most common diaphragmatic abnormalities in infants and is characterized by cyanosis, tachycardia, and asymmetric growth in the chest cavity. By contrast, symptomatic BH is rare in adults. Reduction of the herniated content back into the peritoneal cavity, repair of the hernia, and relief of the intestinal obstruction are important objectives of operative repair. We describe here a case involving a 19-year-old woman with BH who presented with acute paroxysmal left chest pain radiating to the epigastrium. She underwent a mesh-based patch repair and the herniated viscera were returned to the abdominal cavity.

## Case presentation

**Fig. 1:**
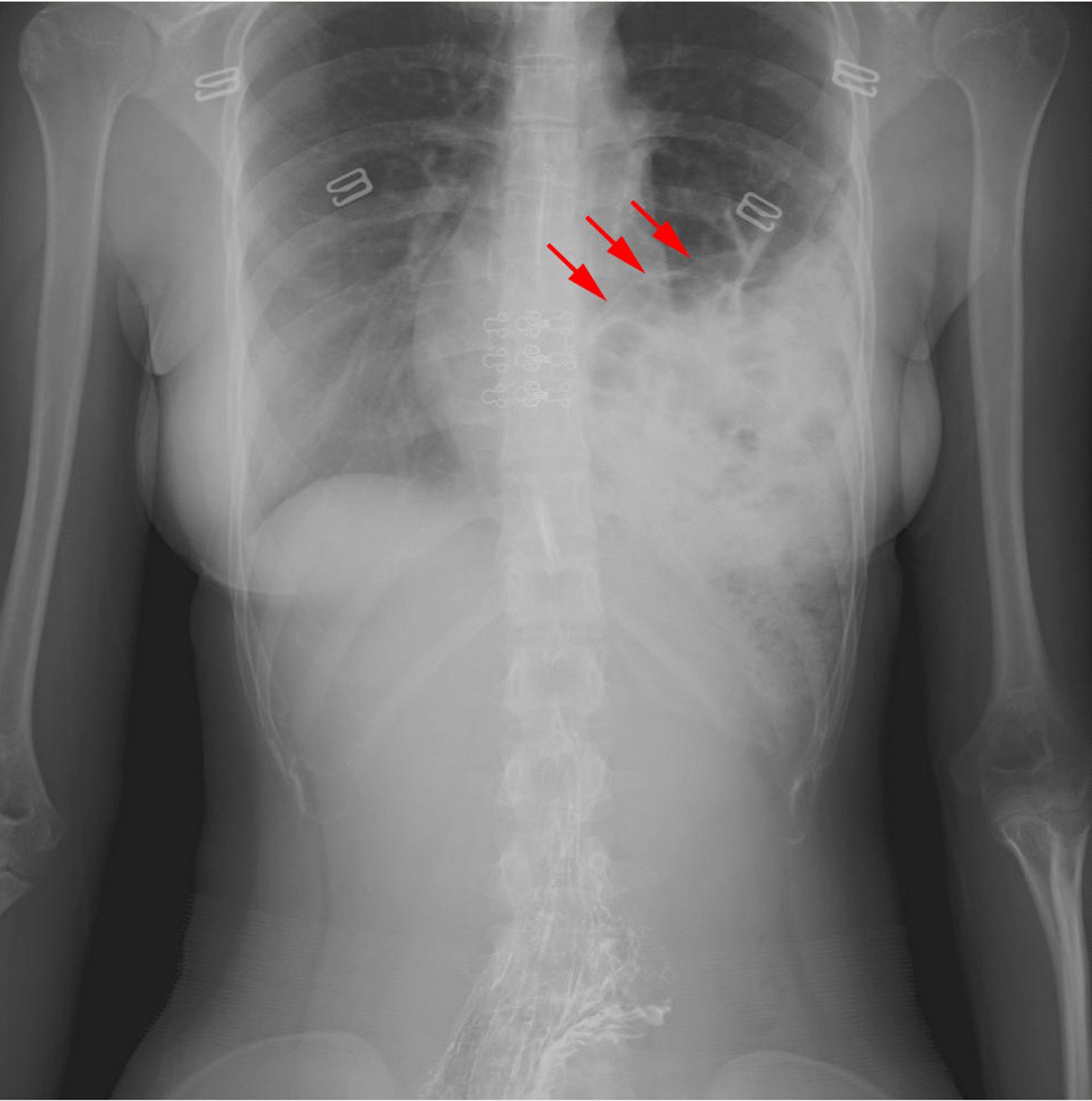
An X-ray showing herniated loops in the left hemithorax, which appeared as multiple lucent shadows and severely reduced lung space (arrow)

**Fig. 2:**
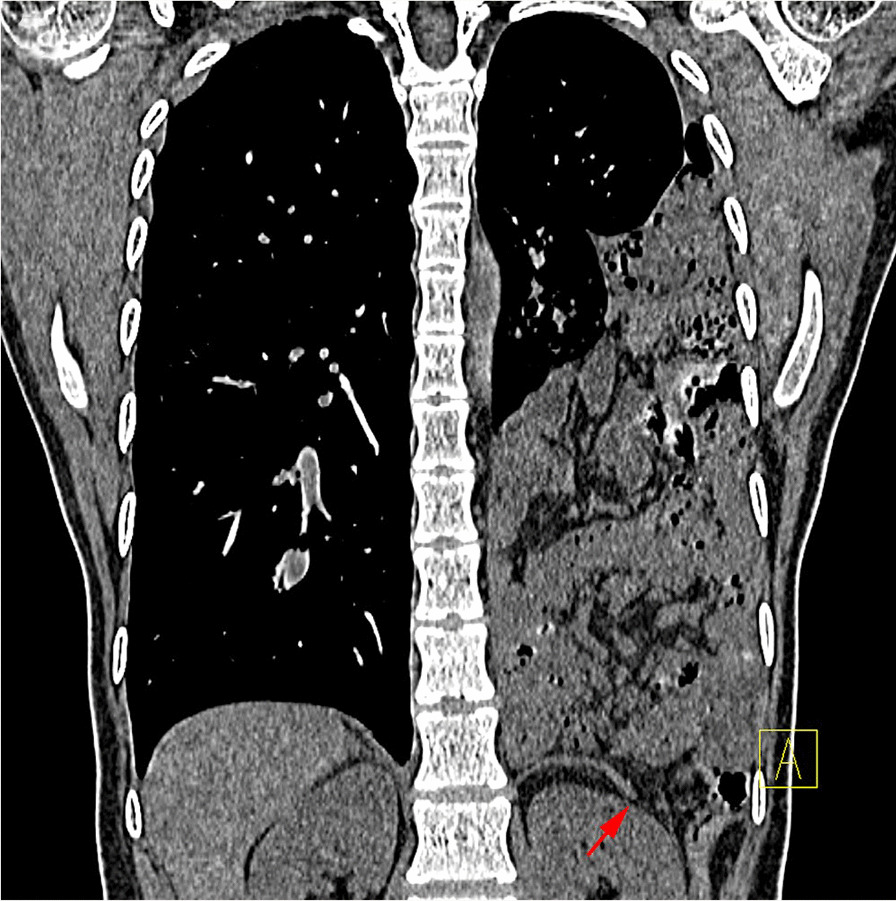
Computed tomography images showing the bowel loops and fat passing through a defect in the posterolateral left hemidiaphragm (arrow)

A 19-year-old woman presented with acute paroxysmal left chest pain radiating to the epigastrium of 4 days duration. She had a 10-year history of exertional dyspnea that was aggravated in the supine position, but no history of chest or abdominal trauma. She was misdiagnosed with a pulmonary abscess at another hospital, and the oral antibiotic treatment was ineffectual. Clinical examination on admission to our hospital revealed bowel sounds and decreased air entry in the left chest and that the abdomen appeared to be scaphoid. No obstructive gastrointestinal symptoms were observed. An X-ray revealed herniated loops of both the small and large intestines in the left hemithorax, which produced multiple lucent shadows and severely reduced lung space (Fig. 1, arrow). Computed tomography (CT) images showed that the bowel loops and fat passed through a defect in the posterolateral left hemidiaphragm (Fig. 2, arrow).

A left posterolateral thoracotomy was performed through the sixth intercostal space. The diaphragmatic defect was found along with herniation of the jejunum, ileum, ileocecal junction, appendix, and ascending and transverse colon. Pulmonary hypoplasia was also seen in the left lower lung lobe. We separated the adhesions around the foramen and within the pleural cavity. A laparotomy was subsequently performed through a median incision. During the operation, anomalies were found in intestinal rotation and fixation, and the total length of the jejunoileum was measured as 750 cm. The herniated viscera were returned to the abdominal cavity.

**Fig. 3:**
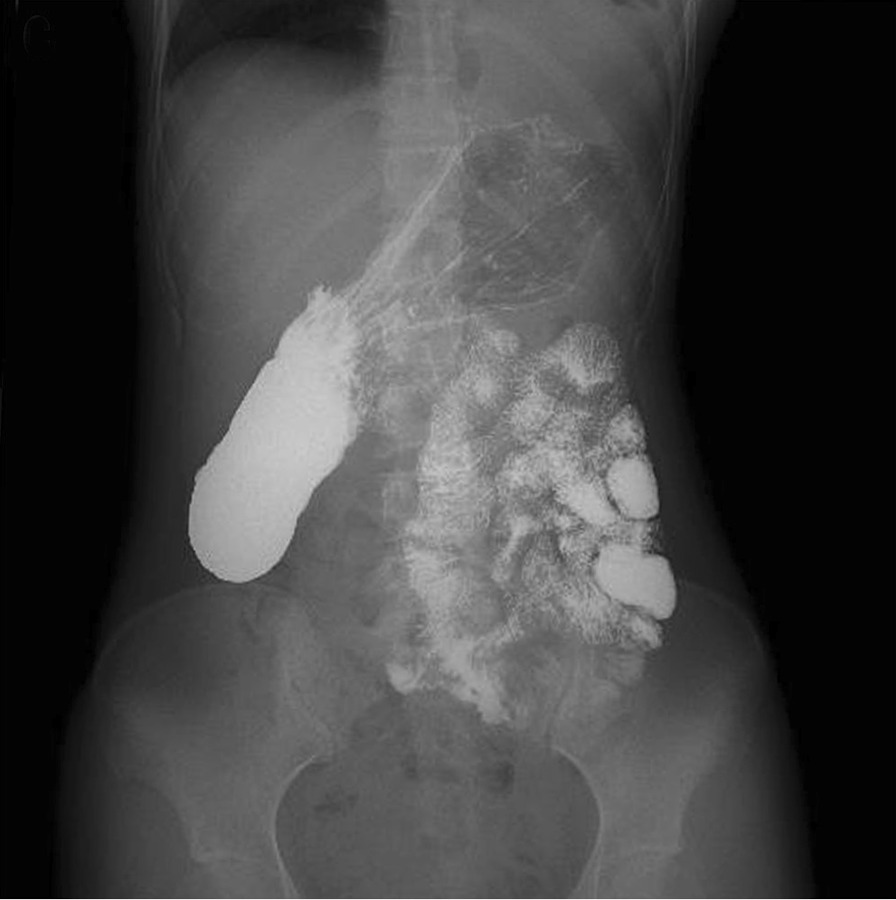
X-ray with soluble iodine contrast at the 1-month follow-up showing the upper gastrointestinal tract and that the repair was satisfactory

**Fig. 4:**
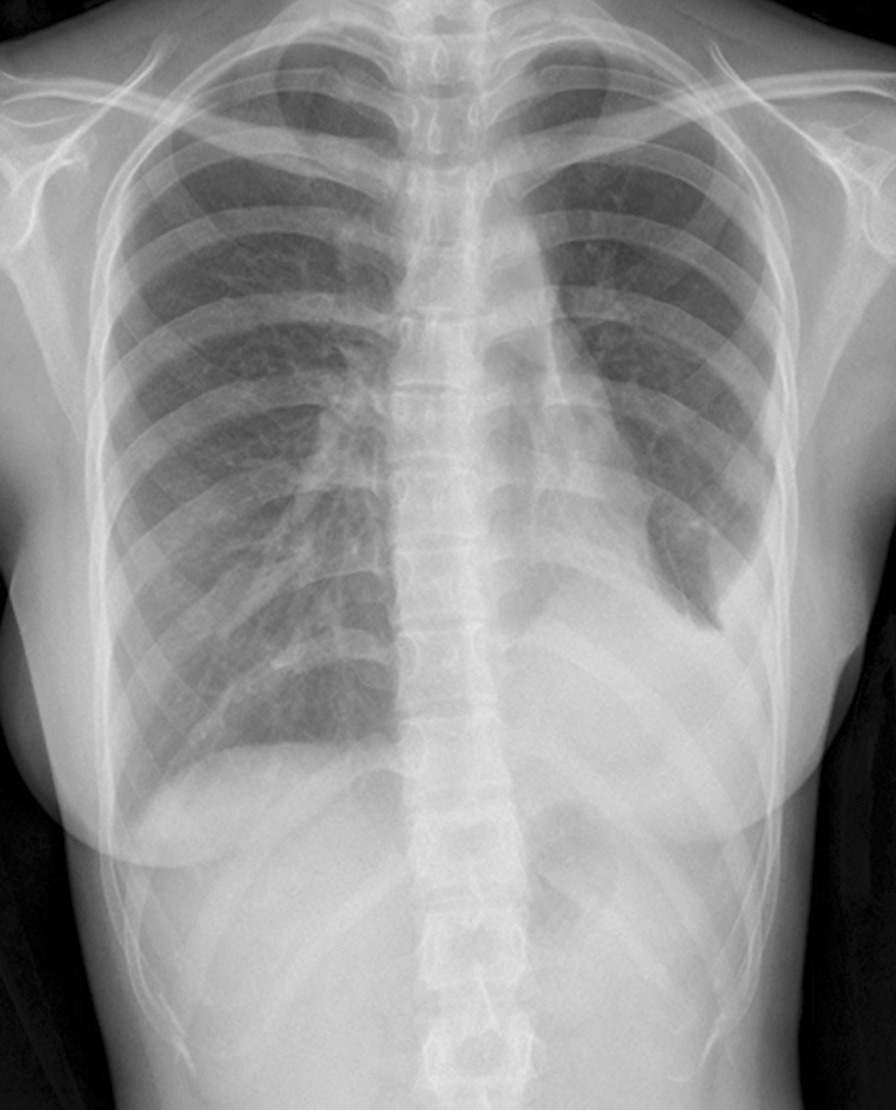
Chest X-ray showing that the left upper lung lobe was completely expanded

We performed a sublay patch repair of the left posterolateral diaphragmatic hernia using a Dacron patch (Balance Medical Technology Co., Ltd., Beijing, China). The procedure lasted for about 3 h. The patient had an uneventful postoperative period. An X-ray of the upper gastrointestinal tract with soluble iodine contrast at the 1-month follow-up showed that the repair was satisfactory (Fig. 3). A chest X-ray revealed that the left upper lung lobe was expanded completely (Fig. 4). After the surgery, the patient’s chest pain and dyspnea were relieved.

## Discussion and conclusions

BH was first described by the Czech anatomist and pathologist, Vincent Alexander Bochdalek (1801–1883), in 1848 [1]. The condition is also known as a congenital posterolateral diaphragmatic hernia or pleuroperitoneal hernia. The abdominal viscera can herniate through into the thorax through the defect in the posterolateral part of the diaphragm. BH is one of the most common diaphragmatic abnormalities in infants and can cause respiratory distress in the newborn baby. The main clinical manifestations are cyanosis, tachycardia, and asymmetric growth in the chest cavity [2]. By contrast, BH is extremely rare in adults; its prevalence is 0.17–6% of all diaphragmatic hernias and it is almost always misdiagnosed.

Patients with BH can be asymptomatic or present with nonspecific gastrointestinal and respiratory signs and symptoms [3]. Other less common congenital hernias of the diaphragm in infants and children include hernias through the central tendon of the diaphragm, paraesophageal hernia, Morgagni hernia, and peritoneal pericardial hernia.

Radiological examination has great clinical value for the discovery of BH and exclusion of other differential diagnoses, especially when BH is an incidental finding or is asymptomatic and involves a small congenital diaphragmatic hernia [4]. Radiological findings can identify diaphragmatic defects, congenital pulmonary malformations, and lesions occupying the intrathoracic space. Surgery is recommended to avoid future strangulation in both symptomatic and asymptomatic adult patients [5]. The primary option is the transabdominal approach, which has lower morbidity compared with thoracotomy. However, a laparoscopic or open laparotomy may not be the best choice for chronically incarcerated giant BHs, in which extensive and severe adhesions are expected in the chest cavity. Therefore, the surgeon should choose an appropriate surgical method based on the situation of each patient.

We have described a rare case of a giant BH in an adult who presented with acute chest pain and dyspnea on exertion. The patient was initially misdiagnosed with a pulmonary abscess. After undergoing a mesh-based patch repair, the patient’s pain and dyspnea were relieved, and good therapeutic outcomes were achieved.

## Data Availability

All data generated or analyzed during this study are included in this published article.

## Abbreviations

BH: Bochdalek hernia
CT: Computed tomography
